# Insights into globalization: comparison of patient characteristics and disease progression among geographic regions in a multinational Alzheimer’s disease clinical program

**DOI:** 10.1186/s13195-018-0443-2

**Published:** 2018-11-24

**Authors:** Jeffrey L. Cummings, Alireza Atri, Clive Ballard, Neli Boneva, Lutz Frölich, José Luis Molinuevo, Lars Lau Raket, Pierre N. Tariot

**Affiliations:** 1 Cleveland Clinic Lou Ruvo Center for Brain Health, 888 W Bonneville Avenue, Las Vegas, NV 89106 USA; 2 Banner Sun Health Research Institute, Sun City, AZ USA; 3 Brigham and Women’s Hospital and Harvard Medical School, Boston, MA USA; 4 University of Exeter Medical School, Exeter, UK; 5 H. Lundbeck A/S, Valby, Denmark; 6 Central Institute of Mental Health, Medical Faculty Mannheim, University of Heidelberg, Heidelberg, Germany; 7 Alzheimer’s Disease and Other Cognitive Disorders Unit, IDIBAPS, Hospital Clinic i Universitari, Barcelona, Spain; 8 Barcelonaβeta Brain Research Center, Pasqual Maragall Foundation, Barcelona, Spain; 9 Banner Alzheimer’s Institute, Phoenix, AZ USA

**Keywords:** Alzheimer’s disease, Humans, Patient selection, Globalization, Clinical trial, Dementia, Disease progression, Cognitive dysfunction, Cognitive decline, Activities of daily living

## Abstract

**Background:**

Globalization of clinical trials has important consequences for trial planning and interpretation. This study investigated heterogeneity in patient characteristics and outcomes among world regions in the global idalopirdine Phase 3 clinical program.

**Methods:**

Data were pooled from three 24-week randomized controlled trials in patients aged ≥ 50 years with mild-to-moderate Alzheimer’s disease (AD) (*n* = 2506). Patients received idalopirdine (10, 30, or 60 mg/day) or placebo, added to cholinesterase inhibitor treatment. Patients were categorized into the following regions: Eastern Europe/Turkey (*n* = 759), Western Europe/Israel (*n* = 709), USA/Canada (*n* = 444), South America/Mexico (*n* = 361), Asia (*n* = 134), and Australia/South Africa (*n* = 99). For each region, operational characteristics, baseline demographic and clinical characteristics, adverse events, and mean change from baseline to week 24 in clinical rating scale scores (placebo group only) were summarized using descriptive statistics.

**Results:**

Completion rates were 0.86–0.90 in all regions. Heterogeneity among global regions was evident. Protocol deviations were twice as common in South America/Mexico as in USA/Canada (2.64 vs 1.35 per patient screened). Educational level ranged from 9.2 years in South America/Mexico to 13.4 years in USA/Canada. *APOE* ε4 carriage was 80.6% in Australia/South Africa, 63.1% in Western Europe/Israel, and < 60% in other regions. Screening Mini–Mental State Examination scores were higher in Eastern Europe/Turkey (18.0) and USA/Canada (17.5) than in other regions (16.9–17.1). Baseline AD Assessment Scale—Cognitive subscale (ADAS-Cog) scores ranged from 24.3 in USA/Canada to 27.2 in South America/Mexico. Baseline AD Cooperative Study—Activities of Daily Living, 23-item version (ADCS-ADL_23_) scores ranged from 58.5 in USA/Canada to 53.5 in Eastern Europe/Turkey. In the placebo group, adverse events were 1.6–1.7 times more common in Western Europe/Israel, USA/Canada, and Australia/South Africa than in Eastern Europe/Turkey. On the ADAS-Cog, Australia/South Africa and Western Europe/Israel showed the most worsening among patients receiving placebo (1.56 and 1.40 points, respectively), whereas South America/Mexico showed an improvement (−0.71 points). All regions worsened on the ADCS-ADL_23_, from −3.21 points in Western Europe/Israel to −0.59 points in Eastern Europe/Turkey.

**Conclusions:**

Regional heterogeneity—in terms of study conduct, patient characteristics, and outcomes—exists, and should be accounted for, when planning and conducting multinational AD clinical trials.

**Trial registration:**

ClinicalTrials.gov, NCT01955161. Registered on 27 September 2013.

ClinicalTrials.gov, NCT02006641. Registered on 5 December 2013.

ClinicalTrials.gov, NCT02006654. Registered on 5 December 2013.

## Background

Alzheimer’s disease (AD) is a highly prevalent, age-related, degenerative brain disease, associated with considerable burden for patients, their family members, caregivers, and society [1]. As the global elderly population increases, the costs associated with AD are also expected to rise [1]. Currently, only two classes of drug are approved for the treatment of AD: cholinesterase inhibitors (ChEIs) and an *N*-methyl-d-aspartate (NMDA) receptor antagonist, memantine [2, 3]. While these agents, and their combination, can provide patients with a symptomatic benefit [2, 4], there remains an urgent need for additional treatment options that can prevent AD, delay its onset, slow its progression, or improve its symptoms [5].

Increasingly, clinical trials of potential new drugs in AD are conducted on an international basis, which allows for faster recruitment of patients, has lower costs, and provides the opportunity to pursue registration and sales in different regions [6, 7]. However, in a global trial, heterogeneity may emerge among geographic regions in terms of clinical characteristics, demographics, comorbidities, clinical measures, and side-effect profiles [6, 8]. This heterogeneity may arise due to differences among regions in pharmacokinetics and pharmacogenetics, the culture of diagnosis and care, the translation of study materials, the experience of study sites, and the attitudes of patients and investigators toward clinical studies [6, 8].

The extent to which these regional factors can impact clinical study outcomes, and to which they should be considered in the design of multinational studies, is unknown. Using data from the multinational Phase 3 clinical program of idalopirdine, the aim of this analysis was to understand the effect of globalization on AD clinical trial design and outcomes by comparing study conduct, patient completion rates, patient characteristics, adverse events, and disease progression across regions.

## Methods

### Study design and patients

Data were pooled from three 24-week, randomized, double-blind, placebo-controlled studies in mild-to-moderate AD: STARSHINE (ClinicalTrials.gov NCT01955161), STARBEAM (ClinicalTrials.gov NCT02006641), and STARBRIGHT (ClinicalTrials.gov NCT02006654). In these studies, treatment with idalopirdine—a selective serotonin 5-HT_6_ receptor antagonist—did not reduce cognitive loss compared with placebo over 24 weeks [9]. For a full description of the designs and outcomes of these studies, see Atri et al. [9].

Each of the three studies had a similar design, comprising: a 2-week screening period during which patient eligibility was monitored by qualified medical staff employed by the sponsor; a 24-week, randomized, double-blind treatment period with regular safety and efficacy assessments; and a 4-week safety follow-up period (or enrollment into an open-label extension study). Patients were enrolled from 34 countries. The studies included male and female outpatients aged ≥ 50 years with a National Institute of Neurological and Communicative Disorders and Stroke–Alzheimer’s Disease and Related Disorders Association (NINCDS-ADRDA) criteria diagnosis of probable AD [10], with a Mini–Mental State Examination (MMSE) score of 12–22 at screening [11], and who had received a therapeutic and stable dose of ChEI for at least 4 months prior to screening (donepezil in STARSHINE and STARBEAM, any ChEI in STARBRIGHT). Patients were excluded if they were taking memantine, had an alternative cause of dementia, had serious non-AD central nervous system or somatic disorders, had clinically significant abnormalities as determined by laboratory testing, or were taking concomitant medications that would interfere with the safety and efficacy assessments. Eligible patients were randomized to double-blind treatment with idalopirdine (fixed doses of 10, 30, or 60 mg/day, depending on the study) or placebo, taken in addition to their standard ChEI treatment.

### Outcome measures

The primary outcome measure of each study was the AD Assessment Scale—Cognitive subscale (ADAS-Cog), an 11-item, objective measure of cognitive impairment scored from 0 to 70, with a higher score indicating more impairment [12]. Key secondary outcome measures in each study were the AD Cooperative Study—Activities of Daily Living, 23-item version (ADCS-ADL_23_) and the AD Cooperative Study—Clinical Global Impression of Change (ADCS-CGIC). The ADCS-ADL_23_ is an informant-rated measure of functional impairment scored from 0 to 78, where a higher score indicates less impairment [13, 14]. The ADCS-CGIC is a clinician-rated measure of: global severity at baseline scored from 1 (normal, not at all ill) to 7 (among the most extremely ill patients); and global change at follow-up scored from 1 (marked improvement) to 7 (marked worsening), where 4 indicates no change [15, 16]. Other secondary outcome measures included the Neuropsychiatric Inventory (NPI) and the MMSE. The NPI is an informant-rated measure of behavioral disturbance (administered by the clinician) scored from 0 to 144, where a higher score indicates more disturbance [17]. The MMSE is an objective measure of cognitive impairment scored from 0 to 30, where a higher score indicates less impairment [11]. External quality oversight methods, including central review of scale administration, were used to achieve consistent and accurate ratings throughout the studies for the ADAS-Cog, ADCS-ADL_23_, ADCS-CGIC, and MMSE.

Safety was assessed via the reporting of adverse events, classified according to the Medical Dictionary for Regulatory Activities (MedDRA) version 19.0.

### Data analysis

For the present post-hoc analysis, patients were categorized into geographic regions according to shared culture, history, geography, and linguistic features, based on the work of Glickman et al. [7] and previous regional studies of AD [18, 19], adjusted for the regions included in the idalopirdine global development program. The resulting six geographic regions were: Eastern Europe/Turkey (comprising Bulgaria, Croatia, Czech Republic, Estonia, Hungary, Lithuania, Poland, Romania, Serbia, Slovakia, Turkey, and Ukraine); Western Europe/Israel (comprising Belgium, Denmark, Finland, France, Germany, Israel, Italy, Portugal, Spain, Switzerland, and United Kingdom); USA/Canada (comprising Canada and USA); South America/Mexico (comprising Argentina, Brazil, Chile, and Mexico); Asia (comprising South Korea, Singapore, and Taiwan); and Australia/South Africa (comprising Australia and South Africa).

For each region, operational characteristics of the clinical program were summarized using descriptive statistics, including the number of sites that screened at least one patient and that randomized at least one patient, the number of patients screened and number of patients randomized per month, and the randomization, completion, and protocol deviation rates.

Baseline demographic and clinical characteristics were summarized descriptively by region for the all-patients-treated set (APTS), defined as all randomized patients who took at least one dose of double-blind medication. Statistical comparisons of baseline variables across all regions were calculated using Kruskal–Wallis tests for the continuous variables, and chi-squared tests for the categorical variables.

The incidences of adverse events, serious adverse events, and deaths were summarized descriptively, per patient in the APTS, by treatment group and by region.

Mean change from baseline to week 24 in the clinical rating scale scores was summarized descriptively using observed cases, by region, in the subpopulation of patients who were randomized to placebo and completed their respective study. For the ADCS-CGIC, which is itself a measure of change from baseline, the mean absolute value at week 24 was summarized using observed cases. Clinical outcomes were investigated in the placebo group rather than the total population to make the results more generalizable, and to prevent the bias that may occur due to a drug’s specific mode of action in relation to the genetic and environmental differences in patients across regions. Statistical comparisons of equality of variances were calculated using Levene tests, and pairwise comparisons between specific regions were calculated using Tukey tests. In case the assumption of equal variances was violated, sensitivity analyses based on Games–Howell post-hoc tests were performed. To evaluate whether regional differences in observed change from baseline to week 24 in the clinical rating scale scores were driven by regional differences in educational level, a linear regression analysis that adjusted for both region and years of education was used as a sensitivity analysis.

Testing was done using a 0.05 significance level (two-sided) with no overall correction for multiple comparisons (regions were corrected for in the Tukey and Games–Howell tests). The statistical analyses were performed using SAS version 9.4 (SAS Institute Inc.).

## Results

### Study conduct

Operational characteristics of the clinical program across regions are presented in Table 1. Western Europe/Israel and USA/Canada had the greatest number of sites (135 and 132, respectively), whereas Asia and Australia/South Africa had the fewest sites (27 and 16, respectively). In Asia and USA/Canada, approximately a third of sites were ‘minimal recruiters’ (randomizing 0–1 patients), whereas in Eastern Europe/Turkey only 3.6% of sites were minimal recruiters. Overall, randomization rates were highest in Eastern Europe/Turkey (0.69 patients randomized per patient screened) and lowest in Asia and USA/Canada (0.51 and 0.48 patients randomized per patient screened, respectively). Completion rates were high in all regions, in the range of 0.86–0.90 completers per patient randomized. Protocol deviation rates were highest in South America/Mexico (2.64 protocol deviations per patient screened) and lowest in USA/Canada (1.35 protocol deviations per patient screened).

**Table 1:** Operational characteristics of the clinical program by region

	Eastern Europe/Turkey	Western Europe/Israel	USA/Canada	South America/Mexico	Asia	Australia/South Africa
Sites that screened at least 1 patient	83	135	132	45	27	16
Randomized 0 patients	1	6	19	1	5	2
Randomized 1 patient only	2	14	23	2	4	2
Randomized 0–1 patients (%)	3 (3.6)	20 (14.8)	42 (31.8)	3 (6.7)	9 (33.3)	4 (25.0)
Patients screened per month^a^	1.23	1.78	1.41	2.03	2.07	1.98
Patients randomized per month^b^	0.84	1.22	0.58	1.30	0.92	0.98
Randomization rate^c^	0.69	0.64	0.48	0.66	0.51	0.54
Completion rate^d^	0.89	0.90	0.89	0.88	0.90	0.86
Protocol deviation rate^e^	1.79	2.33	1.35	2.64	1.47	1.44

^a^Mean of values for each site (number of patients screened/duration of screening period in months). Screening period was defined as the time from first patient screened to last patient screened at the site; sites that screened patients for a period of < 7 days were assigned a screening period of 7 days for this calculation

^b^Mean of values for each site (number of patients randomized/duration of screening period in months). Screening period was defined as the time from first patient screened to last patient screened at the site; sites that screened patients for a period of < 7 days were assigned a screening period of 7 days for this calculation

^c^Mean of values for each site (number of patients randomized/number of patients screened)

^d^Total number of patients completed across all sites/total number of patients randomized across all sites

^e^Mean of values for each site (number of protocol deviations throughout the study/number of patients screened)

### Demographics

A total of 2506 patients received at least one dose of randomized treatment, split across the regions as follows: Eastern Europe/Turkey, 759 (30.3% of the total); Western Europe/Israel, 709 (28.3%); USA/Canada, 444 (17.7%); South America/Mexico, 361 (14.4%); Asia, 134 (5.3%); and Australia/South Africa, 99 (4.0%).

Baseline demographics by region are presented in Table 2. In summary, regional differences were observed for height, weight, body mass index (BMI), educational level, marital status, relationship of caregiver, apolipoprotein E (*APOE*) ε4 carriage, and MMSE score (all *p* < 0.001), but not for age or sex. Specifically, height was lowest in Asia (1.57 m) and South America/Mexico (1.59 m), and comparable in the other regions (1.63–1.64 m). Weight and BMI were lowest in Asia (57.6 kg, 23.4 kg/m^2^), and highest in Australia/South Africa (71.9 kg, 26.8 kg/m^2^) and USA/Canada (71.6 kg, 26.6 kg/m^2^). Educational level was lowest in South America/Mexico (9.2 years) and Asia (9.6 years), and highest in USA/Canada (13.4 years). The proportion of married patients was lowest in Eastern Europe/Turkey (58.2%) and highest in Asia (77.6%). Most commonly, caregivers were a spouse or partner in Western Europe/Israel, USA/Canada, Asia, and Australia/South Africa, and a child in Eastern Europe/Turkey and South America/Mexico. *APOE* ε4 carriage was most common in Australia/South Africa (80.6%), followed by Western Europe/Israel (63.1%), and was < 60% in the other regions, being lowest in Asia (51.5%).

**Table 2:** Baseline demographic and clinical characteristics by region

	Eastern Europe/Turkey (*n* = 759)	Western Europe/Israel (*n* = 709)	USA/Canada (*n* = 444)	South America/Mexico (*n* = 361)	Asia (*n* = 134)	Australia/South Africa (*n* = 99)	*p* value across all regions
**Demographics**							
Age (years), mean (SD)	74.4 (8.2)	73.6 (8.0)	74.3 (9.6)	74.3 (7.7)	73.3 (7.8)	73.9 (7.8)	0.33
Female, *n* (%)	493 (65.0)	449 (63.3)	266 (59.9)	237 (65.7)	87 (64.9)	59 (59.6)	0.46
Height (m), mean (SD)	1.63 (0.09)	1.63 (0.10)	1.64 (0.10)	1.59 (0.09)	1.57 (0.09)	1.63 (0.09)	< 0.0001
Weight (kg), mean (SD)	69.8 (13.2)	67.9 (12.7)	71.6 (15.0)	67.3 (12.2)	57.6 (9.5)	71.9 (15.3)	< 0.0001
BMI (kg/m^2^), mean (SD)	26.1 (4.2)	25.6 (4.0)	26.6 (4.9)	26.5 (4.1)	23.4 (3.0)	26.8 (4.8)	< 0.0001
Education (years), mean (SD)	12.0 (3.3)	10.2 (4.1)	13.4 (3.3)	9.2 (4.3)	9.6 (5.0)	11.5 (3.0)	< 0.0001
Marital status, *n* (%)							< 0.0001
Married/with partner	442 (58.2)	531 (74.9)	294 (66.2)	218 (60.4)	104 (77.6)	74 (74.7)	
Widowed	266 (35.0)	131 (18.5)	94 (21.2)	104 (28.8)	22 (16.4)	14 (14.1)	
Divorced/separated	36 (4.7)	27 (3.8)	38 (8.6)	23 (6.4)	7 (5.2)	8 (8.1)	
Single	15 (2.0)	20 (2.8)	18 (4.1)	16 (4.4)	1 (0.7)	3 (3.0)	
Caregiver, *n* (%)							< 0.0001
Spouse/partner	316 (41.6)	464 (65.4)	257 (57.9)	149 (41.3)	74 (55.2)	62 (62.6)	
Child	333 (43.9)	184 (26.0)	119 (26.8)	164 (45.4)	52 (38.8)	23 (23.2)	
Sibling	14 (1.8)	22 (3.1)	16 (3.6)	9 (2.5)	2 (1.5)	1 (1.0)	
Other family member	54 (7.1)	18 (2.5)	12 (2.7)	17 (4.7)	6 (4.5)	2 (2.0)	
Friend	33 (4.3)	14 (2.0)	27 (6.1)	10 (2.8)	0 (0.0)	8 (8.1)	
Other	9 (1.2)	6 (0.8)^a^	13 (2.9)	12 (3.3)	0 (0.0)	2 (2.0)^a^	
*APOE* ε4 carrier, *n*/*N* (%)^b^	405/745 (54.4)	439/696 (63.1)	239/428 (55.8)	196/342 (57.3)	69/134 (51.5)	75/93 (80.6)	< 0.0001
MMSE (screening), mean (SD)	18.0 (2.9)	17.1 (2.9)	17.5 (3.0)	17.0 (3.0)	16.9 (2.9)	17.1 (3.0)	< 0.0001
MMSE (baseline), mean (SD)	18.4 (2.9)	17.8 (3.3)	18.4 (3.3)	17.8 (3.0)	17.6 (3.2)	17.9 (3.3)	0.0003
MMSE (change from screening to baseline), mean (SD)	0.5 (1.9)	0.7 (2.2)	0.9 (2.3)	0.9 (2.1)	0.7 (2.7)	0.9 (2.3)	0.010
**Clinical characteristics**							
Time since AD diagnosis (years), median (IQR)	1.30 (0.75–2.49)	1.58 (0.89–2.82)	1.90 (0.87–3.61)	2.02 (1.12–3.39)	1.39 (0.68–2.92)	2.20 (1.25–3.66)	< 0.0001
Prestudy treatment duration (years), median (IQR)	1.00 (0.62–1.82)	1.11 (0.65–2.05)	1.31 (0.69–2.68)	1.07 (0.67–1.70)	1.05 (0.58–2.29)	1.26 (0.65–2.42)	< 0.0001
Previously treated with a ChEI other than donepezil,^c^ *n* (%)	18 (2.4)	55 (7.8)	28 (6.3)	24 (6.6)	9 (6.7)	5 (5.1)	< 0.0001
Previously treated with memantine, *n* (%)	57 (7.5)	38 (5.4)	89 (20.0)	65 (18.0)	11 (8.2)	1 (1.0)	< 0.0001
**Clinical rating scale scores, mean (SD)**							
ADAS-Cog	25.9 (8.6)	26.7 (7.9)	24.3 (7.8)	27.2 (8.0)	25.7 (7.1)	25.3 (8.8)	< 0.0001
ADCS-CGIC	3.9 (0.8)	3.9 (0.7)	3.8 (0.7)	3.6 (0.6)	3.7 (0.8)	3.8 (0.7)	< 0.0001
ADCS-ADL_23_	53.5 (13.7)	56.3 (12.9)	58.5 (12.5)	54.5 (13.2)	57.7 (10.8)	56.1 (12.1)	< 0.0001
NPI	10.1 (11.6)	10.1 (10.8)	10.2 (11.6)	10.6 (13.4)	7.2 (7.7)	11.7 (12.9)	0.11

*AD* Alzheimer’s disease, *ADAS-Cog* Alzheimer’s Disease Assessment Scale—Cognitive subscale, *ADCS-ADL*_*23*_ Alzheimer’s Disease Cooperative Study—Activities of Daily Living, 23-item version, *ADCS-CGIC* Alzheimer’s Disease Cooperative Study—Clinical Global Impression of Change, *APOE* apolipoprotein E, *BMI* body mass index, *ChEI* cholinesterase inhibitor, *IQR* interquartile range, *MMSE* Mini–Mental State Examination, *NPI* Neuropsychiatric Inventory, *SD* standard deviation

^a^Not reported for an additional one patient in this region

^b^Number of *APOE* ε4 carriers/total number of patients with an *APOE* ε4 measurement

^c^Donepezil-treated patients only

At screening, the MMSE score was higher (indicating less cognitive impairment) in Eastern Europe/Turkey (18.0) and USA/Canada (17.5) than in the other regions (range 16.9–17.1). A similar pattern was seen at baseline. All regions showed a mean improvement in the MMSE score from screening to baseline. The degree of improvement ranged from 0.5 points in Eastern Europe/Turkey to 0.9 points in USA/Canada, South America/Mexico, and Australia/South Africa. For complete comparisons, see Table 2.

### Clinical characteristics

Clinical characteristics by region are presented in Table 2. Overall, regional differences were observed for time since AD diagnosis, prestudy treatment duration, previous treatment with a ChEI, and previous treatment with memantine (all *p* < 0.0001). Median time since AD diagnosis varied from 1.3 years in Eastern Europe/Turkey to 2.2 years in Australia/South Africa. Median prestudy treatment duration varied from 1.0 year in Eastern Europe/Turkey to 1.3 years in Australia/South Africa and USA/Canada. Previous treatment with a ChEI other than donepezil (among patients currently treated with donepezil as required by protocol) ranged from 2.4% in Eastern Europe/Turkey to 7.8% in Western Europe/Israel. Previous treatment with memantine ranged from 1.0% in Australia/South Africa to 20.0% in USA/Canada.

### Baseline clinical rating scale scores

Regional differences at baseline, also presented in Table 2, were observed for the ADAS-Cog, ADCS-CGIC, and ADCS-ADL_23_ (all *p* < 0.0001), but not for the NPI. The baseline ADAS-Cog score was lowest (less cognitive impairment) in USA/Canada (24.3) and highest (more cognitive impairment) in South America/Mexico (27.2). The baseline ADCS-CGIC (severity) scores were lowest (less severely ill) in South America/Mexico (3.6) and Asia (3.7), and highest (more severely ill) in Eastern Europe/Turkey and Western Europe/Israel (both 3.9). The baseline ADCS-ADL_23_ score was highest (less functional impairment) in USA/Canada (58.5), and lowest (more functional impairment) in Eastern Europe/Turkey (53.5). Although not statistically different across all groups, the baseline NPI score was lower (less behavioral disturbance) in Asia (7.2) than in the other regions (10.1–11.7).

### Adverse events

The incidence of adverse events by region is presented in Table 3. The lowest incidence of adverse events was in Eastern Europe/Turkey, and the highest incidences were generally in Western Europe/Israel, USA/Canada, and Australia/South Africa. In the placebo group, for example, adverse events were 1.6–1.7 times more common in Western Europe/Israel, USA/Canada, and Australia/South Africa than in Eastern Europe/Turkey. The incidence of serious adverse events, and of deaths, was low and comparable in all regions.

**Table 3:** Incidence of adverse events by region

	Eastern Europe/Turkey (*n* = 759)	Western Europe/Israel (*n* = 709)	USA/Canada (*n* = 444)	South America/Mexico (*n* = 361)	Asia (*n* = 134)	Australia/South Africa (*n* = 99)
AEs per patient, mean (SD)						
Placebo	1.01 (1.46) (*n* = 290)	1.64 (1.64) (*n* = 273)	1.63 (2.21) (*n* = 163)	1.24 (1.44) (*n* = 136)	1.48 (1.72) (*n* = 56)	1.76 (2.11) (*n* = 37)
Idalopirdine 10 mg/day	0.83 (1.45) (*n* = 82)	1.89 (1.68) (*n* = 76)	1.69 (2.09) (*n* = 67)	1.05 (1.59) (*n* = 39)	1.24 (1.61) (*n* = 21)	– (*n* = 0)
Idalopirdine 30 mg/day	0.98 (1.35) (*n* = 191)	1.97 (2.06) (*n* = 153)	2.14 (2.44) (*n* = 119)	1.58 (2.06) (*n* = 90)	1.36 (1.97) (*n* = 22)	2.68 (2.00) (*n* = 19)
Idalopirdine 60 mg/day	1.24 (1.82) (*n* = 196)	1.98 (2.11) (*n* = 207)	2.60 (3.01) (*n* = 95)	1.67 (2.21) (*n* = 96)	2.29 (1.98) (*n* = 35)	2.40 (2.21) (*n* = 43)
SAEs per patient, mean (SD)						
Placebo	0.08 (0.35) (*n* = 290)	0.05 (0.25) (*n* = 273)	0.10 (0.67) (*n* = 163)	0.02 (0.19) (*n* = 136)	0.16 (0.85) (*n* = 56)	0.16 (0.50) (*n* = 37)
Idalopirdine 10 mg/day	0.05 (0.22) (*n* = 82)	0.04 (0.26) (*n* = 76)	0.09 (0.34) (*n* = 67)	0.03 (0.16) (*n* = 39)	0.00 (0.00) (*n* = 21)	– (*n* = 0)
Idalopirdine 30 mg/day	0.06 (0.26) (*n* = 191)	0.05 (0.21) (*n* = 153)	0.08 (0.33) (*n* = 119)	0.07 (0.25) (*n* = 90)	0.00 (0.00) (*n* = 22)	0.05 (0.23) (*n* = 19)
Idalopirdine 60 mg/day	0.11 (0.39) (*n* = 196)	0.08 (0.35) (*n* = 207)	0.12 (0.35) (*n* = 95)	0.07 (0.33) (*n* = 96)	0.06 (0.34) (*n* = 35)	0.02 (0.15) (*n* = 43)
Deaths per patient, mean (SD)						
Placebo	0.01 (0.10) (*n* = 290)	0.00 (0.00) (*n* = 273)	0.00 (0.00) (*n* = 163)	0.00 (0.00) (*n* = 136)	0.00 (0.00) (*n* = 56)	0.00 (0.00) (*n* = 37)
Idalopirdine 10 mg/day	0.00 (0.00) (*n* = 82)	0.00 (0.00) (*n* = 76)	0.00 (0.00) (*n* = 67)	0.00 (0.00) (*n* = 39)	0.00 (0.00) (*n* = 21)	– (*n* = 0)
Idalopirdine 30 mg/day	0.01 (0.07) (*n* = 191)	0.01 (0.08) (*n* = 153)	0.00 (0.00) (*n* = 119)	0.00 (0.00) (*n* = 90)	0.00 (0.00) (*n* = 22)	0.00 (0.00) (*n* = 19)
Idalopirdine 60 mg/day	0.01 (0.10) (*n* = 196)	0.00 (0.00) (*n* = 207)	0.00 (0.00) (*n* = 95)	0.01 (0.10) (*n* = 96)	0.00 (0.00) (*n* = 35)	0.00 (0.00) (*n* = 43)

*AE* adverse event, *SAE* serious adverse event, *SD* standard deviation

### Observed change from baseline to week 24 in the placebo group

**Fig. 1:**
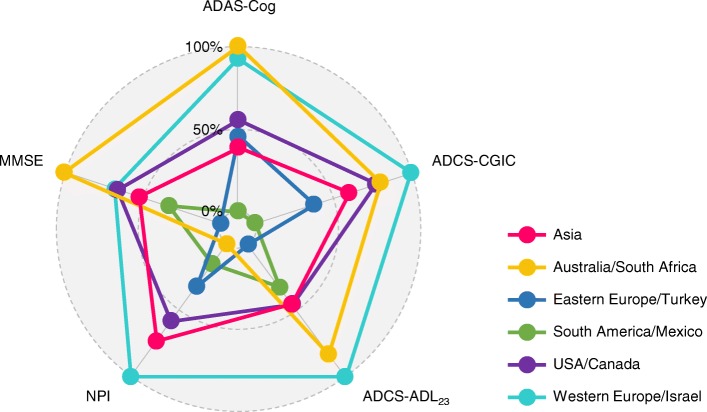
Normalized observed decline from baseline to week 24 across regions in placebo group. 100%, maximal decline on the scale observed across regions; 0%, minimum decline/maximum improvement observed on the scale across regions. *ADAS-Cog* Alzheimer’s Disease Assessment Scale—Cognitive subscale, *ADCS-ADL*_*23*_ Alzheimer’s Disease Cooperative Study—Activities of Daily Living, 23-item version, *ADCS-CGIC* Alzheimer’s Disease Cooperative Study—Clinical Global Impression of Change, *MMSE* Mini–Mental State Examination, *NPI* Neuropsychiatric Inventory

Mean changes in the clinical rating scale scores from baseline to week 24 in the placebo group are presented in Table 4 and Fig. 1. On the ADAS-Cog, all regions showed worsening except for South America/Mexico, which showed an improvement (−0.71). The largest changes from baseline were seen in Australia/South Africa (1.56) and Western Europe/Israel (1.40). Regional differences were also observed on the global outcome (ADCS-CGIC) at week 24: Western Europe/Israel (mean score 4.51), Australia/South Africa (4.41), and USA/Canada (4.39) showed the most worsening, whereas South America/Mexico (3.98) and Eastern Europe/Turkey (4.18) showed no change or the least worsening. Comparison of variance across regions revealed a difference for ADCS-CGIC (*p* < 0.0001), thus violating the assumptions of the Tukey test. A sensitivity analysis assuming unequal variances across regions (Games–Howell post-hoc test) confirmed the significant pairwise differences, except for USA/Canada and South America/Mexico (adjusted *p* = 0.075).

**Table 4:** Observed change from baseline to week 24 by region in the placebo group

	Eastern Europe/Turkey (*n* = 259^a^)	Western Europe/Israel (*n* = 254^b^)	USA/Canada (*n* = 145^c^)	South America/Mexico (*n* = 119^d^)	Asia (*n* = 53)	Australia/South Africa (*n* = 32^e^)	*p* value for equality of variances
ADAS-Cog, mean (SD)	0.32 (6.59)	1.40 (6.24)^SA^	0.55 (5.72)	−0.71 (5.32)^WE^	0.17 (5.28)	1.56 (5.89)	0.29
ADCS-CGIC, mean (SD)^f^	4.18 (0.95)^WE^	4.51 (1.03)^EE,SA^	4.39 (0.90)^SA^	3.98 (1.35)^WE,US^	4.30 (0.85)	4.41 (1.04)	< 0.0001
ADCS-ADL_23_, mean (SD)	−0.59 (7.59)^WE^	−3.21 (7.68)^EE^	−1.78 (8.45)	−1.44 (7.07)	−1.77 (5.71)	−2.76 (8.03)	0.60
NPI, mean (SD)	−1.03 (9.95)	0.45 (9.39)	−0.46 (10.53)	−1.39 (11.42)	−0.13 (6.44)	−1.73 (10.32)	0.66
MMSE, mean (SD)	0.10 (3.08)^WE,US^	−0.79 (2.80)^EE^	−0.77 (3.00)^EE^	−0.34 (2.34)	−0.58 (2.73)	−1.22 (3.03)	0.42

*ADAS-Cog* Alzheimer’s Disease Assessment Scale—Cognitive subscale, *ADCS-ADL*_*23*_ Alzheimer’s Disease Cooperative Study—Activities of Daily Living, 23-item version, *ADCS-CGIC* Alzheimer’s Disease Cooperative Study—Clinical Global Impression of Change, *MMSE* Mini–Mental State Examination, *NPI* Neuropsychiatric Inventory, *SD* standard deviation

Pairwise comparisons: ^EE^*p* < 0.05 versus Eastern Europe/Turkey; ^WE^*p* < 0.05 versus Western Europe/Israel; ^US^*p* < 0.05 versus USA/Canada; ^SA^*p* < 0.05 versus South America/Mexico

^a^Except MMSE, *n* = 260

^b^Except ADCS-CGIC, *n* = 250

^c^Except ADCS-CGIC, *n* = 143

^d^Except ADCS-CGIC, *n* = 117

^e^Except ADCS-ADL_23_ and NPI, *n* = 33

^f^Mean (SD) score at week 24 is presented for ADCS-CGIC. The scale is itself a measure of change from baseline, meaning that change scores are not applicable. Scores > 4 indicate worsening

All regions showed worsening on the functioning outcome (ADCS-ADL_23_); the most worsening was seen in Western Europe/Israel (−3.21), and the least in Eastern Europe/Turkey (−0.59). The behavioral outcome (NPI) showed improvement in all regions (range −0.13 to −1.73) except for Western Europe/Israel (0.45). The MMSE score worsened in all regions (range −0.34 to −1.22) except for Eastern Europe/Turkey (0.10).

When adjustments were made for education as well as region, years of education predicted a faster rate of decline on the ADAS-Cog (0.16 points per added year of education, *p* = 0.0024), and the adjusted difference between Western Europe/Israel and South America/Mexico remained significant. Years of education was not a significant predictor for rate of decline for the other endpoints (all *p* > 0.2).

## Discussion

This analysis demonstrated considerable heterogeneity among geographic regions in the multinational idalopirdine Phase 3 clinical program in AD. Despite the uneven distribution of patients across regions, with small proportions of the total population in Asia and Australia/South Africa, heterogeneity was evident with regard to study conduct, baseline demographic and clinical characteristics, the incidence of adverse events, and the progression of disease in the placebo group. Differences were evident despite all global sites using identical study protocols.

Considering baseline demographic and clinical characteristics, there was a 14-kg difference between the regions with the greatest mean weight (USA/Canada and Australia/South Africa) and the region with the lowest mean weight (Asia). BMI followed a similar regional pattern. Weight and BMI are important considerations in clinical trial design, since they can affect drug distribution and clearance [20] and may affect brain exposure of the administered agent. Patients in USA/Canada had, on average, around 4 years’ more education than patients in South America/Mexico and Asia. A greater level of education has been linked to faster cognitive decline in AD [21, 22], which was observed in this analysis on the ADAS-Cog. The increased rate of decline is thought to arise as a consequence of ‘cognitive reserve’, referring to the ability of some people to tolerate greater neuropathology without developing clinical symptoms [23]. Cognitive reserve is enhanced by education and, therefore, patients with a higher level of education have a greater degree of pathology before clinical symptoms of the disease become manifest [23]. Since the speed of disease progression increases with disease severity, these same patients may deteriorate more rapidly upon diagnosis [21, 22].

Other than Australia/South Africa (80.6%), all regions had a lower proportion of *APOE* ε4 carriers than has been observed in populations of biologically proven AD patients (around 70% in the EXPEDITION3 trial) [24]. This could imply that non-AD patients were recruited into the program, particularly at sites outside Australia and South Africa. Alternatively, these differences could reflect racial differences in the frequency of *APOE* ε4, which is higher in black populations than white populations [25, 26]. The relationship between *APOE* ε4 status and risk of late-onset AD also varies among races, being strongest and most well established in European and Asian populations [27, 28].

Several differences in diagnosis and treatment history were observed across regions, which may reflect differences in culture, societal awareness of AD, and standard of care. The time since AD diagnosis was almost a year longer in Australia/South Africa than in Eastern Europe/Turkey; this could suggest that patients in Eastern Europe/Turkey present to the clinic later in the disease (although MMSE scores at screening were not lower in Eastern Europe/Turkey, indicating a discrepancy between the two approaches to severity), or that study inclusion criteria (such as the requirement for stable donepezil/ChEI treatment) selected for patients with a greater disease duration in some regions.

Considering patient treatment history prior to enrollment, use of a ChEI other than donepezil (among those currently treated with donepezil) was low in all regions (< 10%), whereas prior memantine treatment varied from around 20% in USA/Canada and South America/Mexico to < 10% in all other regions. In the USA, memantine is often initiated as early as ChEIs, despite memantine not being approved for use in mild disease [29]. In the South America/Mexico group, memantine use was mainly driven by patients from Argentina, where reimbursement policy has historically favored memantine such that it has become the most prescribed anti-dementia drug [30]. Of note, the idalopirdine study entry criteria excluded patients currently receiving memantine, which may have contributed to inclusion of more atypical patients in regions where memantine use is common. The reasons why patients stopped memantine treatment prior to the start of the study were not collected.

Baseline clinical rating scale scores were heterogeneous among regions despite the fact that patients were monitored for eligibility during the 2-week screening period, and despite standardization of clinical rating scale usage. There were also differences in patient stability during the screening period, as shown by variation in the change in MMSE score from the screening visit to the baseline visit. Overall, at baseline, patients were least cognitively impaired in USA/Canada, and most impaired in South America/Mexico. Patients in USA/Canada also had the least impairment of functioning at baseline. The greatest impairment in functioning was observed in Eastern Europe/Turkey, which could reflect reporting behaviors of the different informants in this region, with a low proportion of married patients and with more patients being cared for by a child rather than by a spouse or partner. The suitability of activity of daily living (ADL) items to elderly patients differs across regions and can potentially affect functional metrics [31, 32]. Finally, patients showed numerically less behavioral disturbance in Asia than in other regions. Neuropsychiatric symptoms are known to vary among cultures, being influenced by lack of recognition, misinterpretation of certain behaviors, and social stigma [33–36].

Based on analysis of the change in clinical rating scale scores over the first 6 months in the placebo group, the greatest decline (on ADAS-Cog, ADCS-CGIC, and ADCS-ADL_23_) was observed in Western Europe/Israel and Australia/South Africa, and the least decline was observed in Eastern Europe/Turkey and South America/Mexico. The variations may reflect differences in standard of care or accuracy of diagnosis among regions, as well as the proportion of *APOE* ε4 carriers. Such variations in placebo decline will influence the outcome of clinical trials, since low rates of decline among patients receiving placebo will make it more difficult to distinguish a treatment effect.

Overall, two regions stood out in their patterns of characteristics at screening/baseline and their subsequent outcomes/trajectories: Eastern Europe/Turkey and South America/Mexico. Eastern Europe/Turkey had the most impaired functioning (ADCS-ADL_23_) at baseline as well as the least worsening in functioning, which could reflect: the low proportion who were married and differences between informants, as already discussed; cultural differences in the appreciation of the importance of different ADLs; and the positive effect on quality of life and standard of care that can be achieved from enrollment in a clinical trial. This latter point may also be reflected in the observation that Eastern Europe/Turkey was the only region to show improvement on the MMSE over 24 weeks in the placebo group. With regard to South America/Mexico, a similar pattern to that of Eastern Europe/Turkey was observed in terms of baseline ADCS-ADL_23_, change in ADCS-ADL_23_, and proportion of married patients. In addition, South America/Mexico was the only region to show improvement on the ADAS-Cog over 24 weeks (placebo group), despite having the most cognitive impairment at baseline; this may be linked to the lower level of education in this region and differing approaches to diagnosis.

The studies involved in this analysis used a centralized review of scale administration, which reduces administration errors and deviations. Studies not using centralized rating may exhibit more variability.

The incidence of adverse events across treatment arms tended to be higher in Western Europe/Israel, USA/Canada, and Australia/South Africa than in other regions. In the idalopirdine 60 mg/day group, for example, patients in USA/Canada experienced on average one more adverse event than patients in Eastern Europe/Turkey and South America/Mexico. This may have been influenced by differences in baseline medical status (i.e., comorbidities) across regions. Awareness of such variations in adverse event reporting is important for industry sponsors of drug development programs, since reports from Eastern Europe and South America may not reflect the side-effect profile seen in other global regions.

Other large multinational AD clinical programs (e.g., semagacestat and solanezumab) have also shown heterogeneity among geographic regions [18, 19]. Compared with the idalopirdine clinical program, the patterns of heterogeneity were generally similar with regard to baseline demographic and clinical characteristics, with, for example, weight being highest in USA/Canada and Australia/South Africa, and lowest in Asia (and Japan, a separate region in [18]); patient functioning being worst in Eastern Europe and best in USA/Canada; patients showing less behavioral disturbance in Asia (and Japan in [18]) than in other regions; and the incidence of adverse events across treatment arms tending to be higher in USA/Canada and Australia/South Africa than in other regions. Completion rates were high and similar in all regions in the idalopirdine clinical program, whereas significant differences were found among regions in the semagacestat and solanezumab programs. The idalopirdine studies were considerably shorter (24 weeks) than the semagacestat/solanezumab studies (76–80 weeks), so it is possible that differences in completion rates would have emerged given a longer study duration. Solanezumab requires monthly infusions, which may also have influenced participant retention. Furthermore, there may be differences between trials of a symptomatic agent versus a potentially disease-modifying agent.

Heterogeneity may arise for many reasons, including differences in culture and standard of care for patients with AD. A heterogeneous patient population can be advantageous in a clinical program in order to show how an investigative agent affects a real-world AD population. However, it is possible that a heterogeneous patient population may indicate that patients without AD or with atypical AD are being included, making it more difficult to observe a treatment effect, and highlighting the importance in future AD dementia trials of recruiting patients with biomarker evidence of amyloid pathology and neurodegeneration, if proposed research diagnostic criteria are validated.

This analysis of a large clinical trial program is limited by its post-hoc nature, since these studies were not designed to assess regional differences. In addition, homogeneity was not tested within the defined geographic regions, the population sizes were small in the Asia and Australia/South Africa groups, and the analysis did not control for multiple comparisons. These analyses are intended to provide observations of potential importance to those planning, conducting, and interpreting global clinical trials.

## Conclusions

These analyses—conducted on a large, global, clinical trial program—demonstrated that regional heterogeneity exists in multinational AD clinical trials, and that it should be accounted for, whether by limiting or excluding certain countries, increasing sample size to account for the variance, or finding additional means of increasing homogeneity (e.g., biomarkers). Sponsors must be familiar with regional differences in order to appropriately plan and power studies of global programs. Differences in training and vigilance may also be required among regions.

## Abbreviations

AD: Alzheimer’s disease
ADAS-Cog: Alzheimer’s Disease Assessment Scale—Cognitive subscale
ADCS-ADL_23_: Alzheimer’s Disease Cooperative Study—Activities of Daily Living, 23-item version
ADCS-CGIC: Alzheimer’s Disease Cooperative Study—Clinical Global Impression of Change
ADL: Activity of daily living
AE: Adverse event
APOE: Apolipoprotein E
APTS: All-patients-treated set
BMI: Body mass index
ChEI: Cholinesterase inhibitor
IQR: Interquartile range
MedDRA: Medical Dictionary for Regulatory Activities
MMSE: Mini–Mental State Examination
NINCDS-ADRDA: National Institute of Neurological and Communicative Disorders and Stroke–Alzheimer’s Disease and Related Disorders Association
NMDA: *N*-methyl-d-aspartate
NPI: Neuropsychiatric Inventory
SAE: Serious adverse event
SD: Standard deviation
